# 1-[5-(3,5-Di­meth­oxy­phen­yl)-3-(2-meth­oxy­phen­yl)-4,5-di­hydro-1*H*-pyrazol-1-yl]ethanone

**DOI:** 10.1107/S2414314621000961

**Published:** 2021-01-29

**Authors:** Miri Yoo, Dongsoo Koh

**Affiliations:** a Department of Applied Chemistry, Dongduk Women’s University, Seoul 136-714, Republic of Korea; University of Aberdeen, Scotland

**Keywords:** crystal structure, pyrazolines, C—H⋯O hydrogen bonds

## Abstract

In the title compound, C_20_H_22_N_2_O_4_, two benzene rings bearing meth­oxy substituents are connected by a central acetyl­pyrazoline ring: the dihedral angle between the benzene rings is 83.7 (1)°. In the crystal, pairwise C—H⋯O hydrogen bonds generate inversion dimers and additional weak C—H⋯O inter­actions link the dimers into chains propagating along the *c*-axis direction.

## Abstract 2

In the title compound, two benzene rings bearing meth­oxy substituents are connected by central acetyl­pyrazoline ring: the dihedral angle between the benzene rings is 83.7 (1)°. In the crystal, pairwise C—H⋯O hydrogen bonds generate inversion dimers and additional weak C—H⋯O inter­actions link the dimers into chains propagating along the *c*-axis direction.

## Graphical abstract

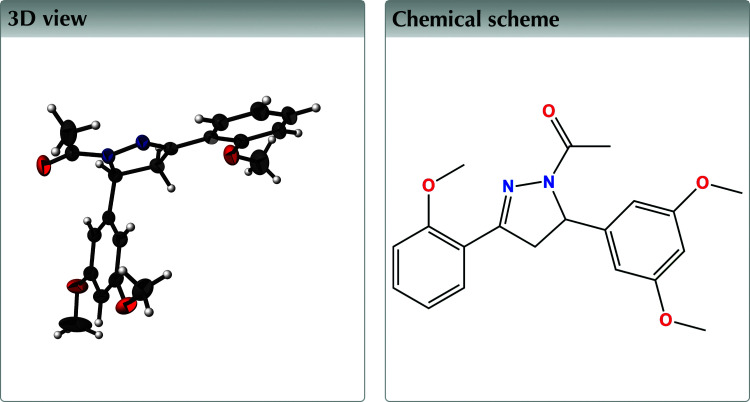

## Structure description

Pyrazolines show a broad spectrum of biological activities including anti­cancer (Matiadis & Sagnou, 2020 ▸), Alzheimer drugs (Neudorfer *et al.*, 2014 ▸) and the dual function of anti­malarial and anti­microbial activities (Mishra, *et al.*, 2017 ▸). According to a recent review, pyrazolines have also demonstrated versatile applications in bio-imaging and sensing (Varghese *et al.*, 2017 ▸). In a continuation of our studies of pyrazolines that show a broad range of biological activities (Jung *et al.* 2015 ▸), the title compound was synthesized and its crystal structure was determined.

**Figure 1:**
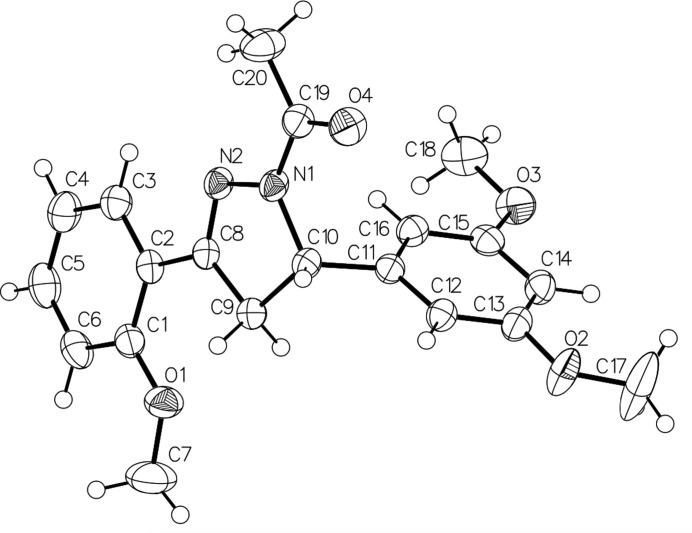
The mol­ecular structure of the title compound, showing the atom-labelling scheme, with displacement ellipsoids drawn at the 30% probability level.

The mol­ecular structure of the title compound is shown in Fig. 1 ▸. Atom C10 has an *S* configuration in the arbitrarily chosen asymmetric unit but crystal symmetry generates a racemic mixture. The central pyrazoline ring (N1/N2/C8–C10) connects the two benzene rings (C1–C6 and C11–C16) at carbon atoms 8 and 10, respectively. The dihedral angle between the pyrazoline ring and the C1–C6 benzene ring is 6.25 (2)°, indicating that the rings are close to coplanar. On the other hand, the dihedral angle formed by the pyrazoline ring and the C11–C16 benzene ring is 83.9 (3)°, which indicates that these two rings are almost orthogonal to each other. The dihedral angle between the benzene rings is 83.7 (1)°. There are three meth­oxy groups, which are attached to carbon-atom C1 of the first benzene ring and C13 and C15 of the second. The C18 atom of the meth­oxy group at C15 is essentially co-planar with the benzene ring [C18—O3—C15—C16 = 0.5 (2)°], whereas the C7 and C17 atoms of the meth­oxy groups at C1 and C13 are slightly twisted from the corresponding ring plane with torsion angles C6—C1—O1—C7 = 6.1 (2)° and C14—C13—O2—C17 = −2.7 (3)°, respectively. The acetyl group attached to the pyrazoline ring lies in almost the same plane as the ring [C20—C19—N1—N2 = 0.9 (2)°].

**Figure 2:**
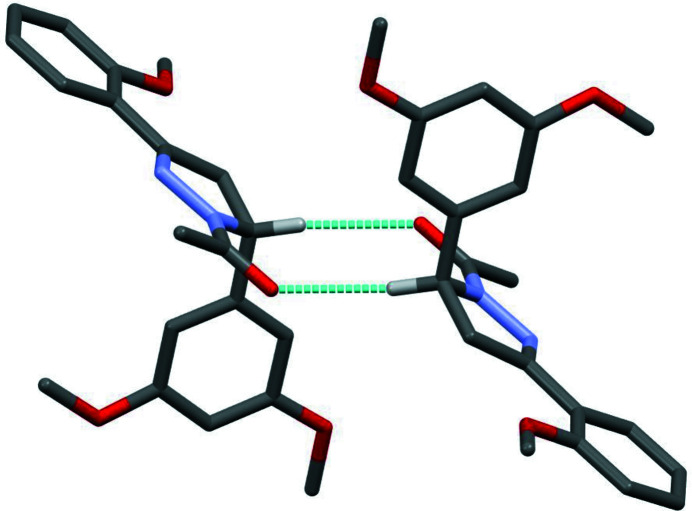
A view of the dimer formed by pairwise C—H⋯O hydrogen bonds (dashed lines) in the crystal structure of the title compound. For clarity, only those H atoms involved in hydrogen bonding are shown.

**Figure 3:**
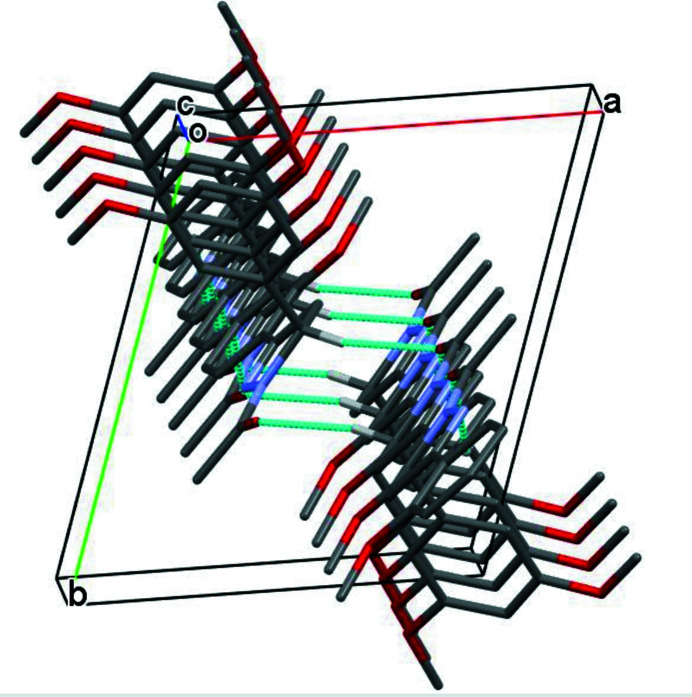
Part of the crystal structure with hydrogen bonds shown as dashed lines. For clarity, only those H atoms involved in hydrogen bonding are shown.

In the crystal, pairs of C10—H10⋯O4 hydrogen bonds generate inversion dimers (Table 1 ▸, Fig. 2 ▸) featuring 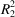 (10) loops and another pair of C—H⋯O hydrogen bonds links the dimers into chains propagating along the *c*-axis direction (Fig. 3 ▸).

## Synthesis and crystallization

To a solution of 2-meth­oxy­aceto­phenone (600 mg, 4 mmol) in 50 ml of ethanol was added 3,5-di­meth­oxy­benzaldehyde (830 mg, 5 mmol) and the temperature was adjusted to around 277 K in an ice-bath. To the cooled reaction mixture were added 5 ml of 40% aqueous KOH solution, and the reaction mixture was stirred at room temperature for 5 h. This mixture was poured into iced water (100 ml) and was acidified (pH = 3) with 2 *N* HCl solution to give a precipitate. Filtration and washing with water afforded a crude solid of a chalcone compound, which was recrystallized from ethanol solution. To a solution of the chalcone compound (2 mmol, 596 mg) in 20 ml of anhydrous ethanol was added excess hydrazine monohydrate (0.6 ml of 64–65% solution, 7 mmol) and the solution was refluxed at 362 K for 3 h. The reaction mixture was cooled to room temperature to produce a solid. This solid was recrystallized from an ethanol solution to obtain single crystals of the title compound (m.p. 436–437 K, yield; 62%).

## Refinement

Crystal data, data collection and structure refinement details are summarized in Table 2 ▸.

## Supplementary Material

Crystal structure: contains datablock(s) I. DOI: 10.1107/S2414314621000961/hb4374sup1.cif

Structure factors: contains datablock(s) I. DOI: 10.1107/S2414314621000961/hb4374Isup2.hkl

Supporting information file. DOI: 10.1107/S2414314621000961/hb4374Isup3.cml

CCDC reference: 2058998

Additional supporting information:  crystallographic information; 3D view; checkCIF report

## Figures and Tables

**Table 1:** Hydrogen-bond geometry (Å, °)

*D*—H⋯*A*	*D*—H	H⋯*A*	*D*⋯*A*	*D*—H⋯*A*
C10—H10⋯O4^i^	0.99	2.46	3.4340 (18)	167
C5—H5⋯O4^ii^	0.94	2.59	3.309 (2)	134

Symmetry codes: (i) 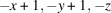 ; (ii) 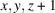 .

**Table 2:** Experimental details

Crystal data	
Chemical formula	C_20_H_22_N_2_O_4_
*M*_r_	354.39
Crystal system, space group	Triclinic, *P* 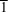
Temperature (K)	223
*a*, *b*, *c* (Å)	8.7181 (2), 10.7230 (3), 11.2096 (3)
α, β, γ (°)	113.0348 (10), 91.4395 (12), 107.4022 (11)
*V* (Å^3^)	908.09 (4)
*Z*	2
Radiation type	Mo *K*α
μ (mm^−1^)	0.09
Crystal size (mm)	0.17 × 0.16 × 0.12

Data collection	
Diffractometer	PHOTON 100 CMOS
Absorption correction	Multi-scan (*SADABS*; Bruker, 2012 ▸)
*T*_min_, *T*_max_	0.643, 0.746
No. of measured, independent and observed [*I* > 2σ(*I*)] reflections	39068, 4541, 3338
*R*_int_	0.045
(sin θ/λ)_max_ (Å^−1^)	0.669

Refinement	
*R*[*F* ^2^ > 2σ(*F* ^2^)], *wR*(*F* ^2^), *S*	0.046, 0.126, 1.05
No. of reflections	4541
No. of parameters	239
H-atom treatment	H-atom parameters constrained
Δρ_max_, Δρ_min_ (e Å^−3^)	0.31, −0.21

Computer programs: *APEX2* and *SAINT* (Bruker, 2012 ▸), *SHELXTL* (Sheldrick, 2008 ▸) and *SHELXL2014/7* (Sheldrick, 2015 ▸).

## full crystallographic data

### Crystal data

**Table:**

C_20_H_22_N_2_O_4_	*Z* = 2
*M**_r_* = 354.39	*F*(000) = 376
Triclinic, *P*1	*D*_x_ = 1.296 Mg m^−^^3^
*a* = 8.7181 (2) Å	Mo *K*α radiation, λ = 0.71073 Å
*b* = 10.7230 (3) Å	Cell parameters from 9964 reflections
*c* = 11.2096 (3) Å	θ = 2.3–28.3°
α = 113.0348 (10)°	µ = 0.09 mm^−^^1^
β = 91.4395 (12)°	*T* = 223 K
γ = 107.4022 (11)°	Block, colourless
*V* = 908.09 (4) Å^3^	0.17 × 0.16 × 0.12 mm

### Data collection

**Table:**

PHOTON 100 CMOS diffractometer	3338 reflections with *I* > 2σ(*I*)
φ and ω scans	*R*_int_ = 0.045
Absorption correction: multi-scan (SADABS; Bruker, 2012)	θ_max_ = 28.4°, θ_min_ = 2.2°
*T*_min_ = 0.643, *T*_max_ = 0.746	*h* = −11→11
39068 measured reflections	*k* = −14→14
4541 independent reflections	*l* = −14→14

### Refinement

**Table:**

Refinement on *F*^2^	Primary atom site location: structure-invariant direct methods
Least-squares matrix: full	Hydrogen site location: inferred from neighbouring sites
*R*[*F*^2^ > 2σ(*F*^2^)] = 0.046	H-atom parameters constrained
*wR*(*F*^2^) = 0.126	*w* = 1/[σ^2^(*F*_o_^2^) + (0.050*P*)^2^ + 0.3528*P*] where *P* = (*F*_o_^2^ + 2*F*_c_^2^)/3
*S* = 1.05	(Δ/σ)_max_ < 0.001
4541 reflections	Δρ_max_ = 0.31 e Å^−^^3^
239 parameters	Δρ_min_ = −0.21 e Å^−^^3^
0 restraints	

### Special details

**Table:**

Geometry. All esds (except the esd in the dihedral angle between two l.s. planes) are estimated using the full covariance matrix. The cell esds are taken into account individually in the estimation of esds in distances, angles and torsion angles; correlations between esds in cell parameters are only used when they are defined by crystal symmetry. An approximate (isotropic) treatment of cell esds is used for estimating esds involving l.s. planes.

### Fractional atomic coordinates and isotropic or equivalent isotropic displacement parameters (Å^2^)

**Table:**

	*x*	*y*	*z*	*U*_iso_*/*U*_eq_	
C1	0.33313 (18)	0.31123 (16)	0.49923 (14)	0.0328 (3)	
C2	0.28231 (17)	0.40413 (15)	0.46044 (13)	0.0290 (3)	
C3	0.20042 (19)	0.48741 (17)	0.54478 (14)	0.0349 (3)	
H3	0.1650	0.5503	0.5209	0.042*	
C4	0.1704 (2)	0.47940 (19)	0.66259 (16)	0.0423 (4)	
H4	0.1143	0.5357	0.7174	0.051*	
C5	0.2233 (2)	0.38832 (19)	0.69949 (16)	0.0439 (4)	
H5	0.2041	0.3837	0.7800	0.053*	
C6	0.3037 (2)	0.30458 (18)	0.61869 (15)	0.0391 (4)	
H6	0.3390	0.2426	0.6441	0.047*	
O1	0.41126 (15)	0.23045 (13)	0.41477 (11)	0.0445 (3)	
C7	0.4509 (3)	0.1255 (2)	0.4431 (2)	0.0658 (6)	
H7A	0.5253	0.1720	0.5256	0.099*	
H7B	0.5023	0.0745	0.3733	0.099*	
H7C	0.3525	0.0580	0.4498	0.099*	
C8	0.30805 (17)	0.41521 (15)	0.33494 (13)	0.0274 (3)	
C9	0.38469 (18)	0.32990 (16)	0.22753 (13)	0.0304 (3)	
H9A	0.3314	0.2262	0.1994	0.037*	
H9B	0.5013	0.3553	0.2562	0.037*	
C10	0.35565 (17)	0.37553 (15)	0.11689 (13)	0.0285 (3)	
H10	0.4592	0.4092	0.0863	0.034*	
N1	0.29693 (15)	0.49645 (12)	0.18619 (11)	0.0297 (3)	
N2	0.26272 (15)	0.50766 (13)	0.31007 (11)	0.0298 (3)	
C11	0.22958 (17)	0.25555 (14)	0.00294 (13)	0.0277 (3)	
C12	0.27974 (18)	0.17696 (15)	−0.10909 (14)	0.0304 (3)	
H12	0.3907	0.2019	−0.1172	0.036*	
C13	0.16453 (19)	0.06023 (16)	−0.21028 (14)	0.0339 (3)	
C14	0.00146 (19)	0.02227 (16)	−0.20007 (15)	0.0358 (3)	
H14	−0.0754	−0.0568	−0.2686	0.043*	
C15	−0.04762 (18)	0.10307 (15)	−0.08643 (15)	0.0325 (3)	
C16	0.06497 (18)	0.21904 (15)	0.01496 (14)	0.0312 (3)	
H16	0.0309	0.2729	0.0914	0.037*	
O2	0.22721 (16)	−0.01188 (13)	−0.31699 (11)	0.0514 (3)	
C17	0.1152 (3)	−0.1365 (3)	−0.4194 (2)	0.0962 (10)	
H17A	0.0575	−0.2030	−0.3836	0.144*	
H17B	0.1739	−0.1825	−0.4857	0.144*	
H17C	0.0378	−0.1091	−0.4586	0.144*	
O3	−0.21162 (13)	0.05856 (12)	−0.08562 (12)	0.0446 (3)	
C18	−0.2693 (2)	0.1386 (2)	0.0275 (2)	0.0499 (4)	
H18A	−0.2214	0.1346	0.1043	0.075*	
H18B	−0.3870	0.0978	0.0156	0.075*	
H18C	−0.2387	0.2380	0.0394	0.075*	
C19	0.26834 (19)	0.58262 (16)	0.13306 (14)	0.0332 (3)	
O4	0.29910 (15)	0.56660 (13)	0.02318 (11)	0.0439 (3)	
C20	0.1993 (3)	0.6953 (2)	0.21300 (18)	0.0534 (5)	
H20A	0.2756	0.7898	0.2308	0.080*	
H20B	0.1805	0.6879	0.2953	0.080*	
H20C	0.0971	0.6813	0.1647	0.080*	

### Atomic displacement parameters (Å^2^)

**Table:**

	*U*^11^	*U*^22^	*U*^33^	*U*^12^	*U*^13^	*U*^23^
C1	0.0332 (8)	0.0326 (7)	0.0298 (7)	0.0082 (6)	0.0000 (6)	0.0126 (6)
C2	0.0282 (7)	0.0294 (7)	0.0249 (7)	0.0047 (6)	0.0007 (5)	0.0106 (6)
C3	0.0377 (8)	0.0367 (8)	0.0295 (7)	0.0119 (7)	0.0055 (6)	0.0134 (6)
C4	0.0450 (9)	0.0474 (9)	0.0305 (8)	0.0142 (8)	0.0106 (7)	0.0130 (7)
C5	0.0472 (10)	0.0525 (10)	0.0286 (8)	0.0071 (8)	0.0045 (7)	0.0209 (7)
C6	0.0423 (9)	0.0426 (9)	0.0341 (8)	0.0087 (7)	−0.0006 (7)	0.0222 (7)
O1	0.0607 (8)	0.0464 (7)	0.0405 (6)	0.0301 (6)	0.0114 (6)	0.0233 (5)
C7	0.0888 (16)	0.0660 (13)	0.0723 (14)	0.0512 (13)	0.0194 (12)	0.0398 (12)
C8	0.0274 (7)	0.0261 (7)	0.0240 (6)	0.0057 (5)	0.0009 (5)	0.0083 (5)
C9	0.0329 (7)	0.0315 (7)	0.0267 (7)	0.0121 (6)	0.0036 (6)	0.0110 (6)
C10	0.0314 (7)	0.0281 (7)	0.0246 (7)	0.0100 (6)	0.0060 (6)	0.0095 (6)
N1	0.0396 (7)	0.0269 (6)	0.0228 (6)	0.0113 (5)	0.0067 (5)	0.0104 (5)
N2	0.0375 (7)	0.0291 (6)	0.0229 (6)	0.0112 (5)	0.0062 (5)	0.0109 (5)
C11	0.0334 (7)	0.0258 (7)	0.0256 (7)	0.0097 (6)	0.0042 (6)	0.0126 (6)
C12	0.0330 (7)	0.0290 (7)	0.0279 (7)	0.0088 (6)	0.0060 (6)	0.0118 (6)
C13	0.0429 (9)	0.0284 (7)	0.0270 (7)	0.0105 (6)	0.0058 (6)	0.0093 (6)
C14	0.0397 (8)	0.0266 (7)	0.0341 (8)	0.0055 (6)	−0.0026 (6)	0.0105 (6)
C15	0.0310 (7)	0.0285 (7)	0.0403 (8)	0.0074 (6)	0.0028 (6)	0.0187 (6)
C16	0.0339 (8)	0.0306 (7)	0.0303 (7)	0.0117 (6)	0.0073 (6)	0.0132 (6)
O2	0.0524 (7)	0.0409 (7)	0.0345 (6)	0.0056 (6)	0.0106 (5)	−0.0036 (5)
C17	0.0702 (16)	0.0801 (17)	0.0559 (14)	−0.0046 (13)	0.0155 (12)	−0.0327 (12)
O3	0.0308 (6)	0.0366 (6)	0.0593 (8)	0.0054 (5)	0.0046 (5)	0.0176 (6)
C18	0.0377 (9)	0.0509 (10)	0.0661 (12)	0.0142 (8)	0.0189 (9)	0.0296 (10)
C19	0.0402 (8)	0.0308 (7)	0.0293 (7)	0.0099 (6)	0.0061 (6)	0.0148 (6)
O4	0.0623 (8)	0.0473 (7)	0.0328 (6)	0.0228 (6)	0.0169 (5)	0.0239 (5)
C20	0.0868 (15)	0.0509 (11)	0.0473 (10)	0.0421 (11)	0.0271 (10)	0.0305 (9)

### Geometric parameters (Å, º)

**Table:**

C1—O1	1.3617 (18)	N1—N2	1.3940 (15)
C1—C6	1.395 (2)	C11—C12	1.3778 (19)
C1—C2	1.404 (2)	C11—C16	1.395 (2)
C2—C3	1.398 (2)	C12—C13	1.394 (2)
C2—C8	1.4746 (19)	C12—H12	0.9400
C3—C4	1.383 (2)	C13—O2	1.3744 (18)
C3—H3	0.9400	C13—C14	1.378 (2)
C4—C5	1.383 (2)	C14—C15	1.394 (2)
C4—H4	0.9400	C14—H14	0.9400
C5—C6	1.375 (2)	C15—O3	1.3668 (18)
C5—H5	0.9400	C15—C16	1.382 (2)
C6—H6	0.9400	C16—H16	0.9400
O1—C7	1.412 (2)	O2—C17	1.421 (2)
C7—H7A	0.9700	C17—H17A	0.9700
C7—H7B	0.9700	C17—H17B	0.9700
C7—H7C	0.9700	C17—H17C	0.9700
C8—N2	1.2891 (18)	O3—C18	1.427 (2)
C8—C9	1.5090 (19)	C18—H18A	0.9700
C9—C10	1.5411 (19)	C18—H18B	0.9700
C9—H9A	0.9800	C18—H18C	0.9700
C9—H9B	0.9800	C19—O4	1.2236 (17)
C10—N1	1.4749 (17)	C19—C20	1.497 (2)
C10—C11	1.5141 (19)	C20—H20A	0.9700
C10—H10	0.9900	C20—H20B	0.9700
N1—C19	1.3534 (19)	C20—H20C	0.9700
			
O1—C1—C6	123.01 (14)	C8—N2—N1	108.20 (11)
O1—C1—C2	116.47 (13)	C12—C11—C16	120.38 (13)
C6—C1—C2	120.52 (14)	C12—C11—C10	119.43 (12)
C3—C2—C1	117.68 (13)	C16—C11—C10	120.06 (12)
C3—C2—C8	119.07 (13)	C11—C12—C13	119.39 (14)
C1—C2—C8	123.24 (13)	C11—C12—H12	120.3
C4—C3—C2	121.49 (15)	C13—C12—H12	120.3
C4—C3—H3	119.3	O2—C13—C14	124.09 (14)
C2—C3—H3	119.3	O2—C13—C12	114.83 (14)
C3—C4—C5	119.82 (15)	C14—C13—C12	121.07 (14)
C3—C4—H4	120.1	C13—C14—C15	118.92 (14)
C5—C4—H4	120.1	C13—C14—H14	120.5
C6—C5—C4	120.19 (14)	C15—C14—H14	120.5
C6—C5—H5	119.9	O3—C15—C16	124.38 (14)
C4—C5—H5	119.9	O3—C15—C14	114.83 (13)
C5—C6—C1	120.29 (15)	C16—C15—C14	120.79 (14)
C5—C6—H6	119.9	C15—C16—C11	119.44 (13)
C1—C6—H6	119.9	C15—C16—H16	120.3
C1—O1—C7	118.67 (14)	C11—C16—H16	120.3
O1—C7—H7A	109.5	C13—O2—C17	116.73 (15)
O1—C7—H7B	109.5	O2—C17—H17A	109.5
H7A—C7—H7B	109.5	O2—C17—H17B	109.5
O1—C7—H7C	109.5	H17A—C17—H17B	109.5
H7A—C7—H7C	109.5	O2—C17—H17C	109.5
H7B—C7—H7C	109.5	H17A—C17—H17C	109.5
N2—C8—C2	118.77 (12)	H17B—C17—H17C	109.5
N2—C8—C9	113.69 (12)	C15—O3—C18	117.33 (13)
C2—C8—C9	127.53 (12)	O3—C18—H18A	109.5
C8—C9—C10	102.65 (11)	O3—C18—H18B	109.5
C8—C9—H9A	111.2	H18A—C18—H18B	109.5
C10—C9—H9A	111.2	O3—C18—H18C	109.5
C8—C9—H9B	111.2	H18A—C18—H18C	109.5
C10—C9—H9B	111.2	H18B—C18—H18C	109.5
H9A—C9—H9B	109.2	O4—C19—N1	119.79 (14)
N1—C10—C11	111.67 (12)	O4—C19—C20	122.44 (14)
N1—C10—C9	101.24 (10)	N1—C19—C20	117.77 (13)
C11—C10—C9	112.00 (11)	C19—C20—H20A	109.5
N1—C10—H10	110.5	C19—C20—H20B	109.5
C11—C10—H10	110.5	H20A—C20—H20B	109.5
C9—C10—H10	110.5	C19—C20—H20C	109.5
C19—N1—N2	122.73 (12)	H20A—C20—H20C	109.5
C19—N1—C10	124.12 (11)	H20B—C20—H20C	109.5
N2—N1—C10	113.02 (11)		
			
O1—C1—C2—C3	179.43 (13)	C19—N1—N2—C8	−177.84 (13)
C6—C1—C2—C3	−0.7 (2)	C10—N1—N2—C8	6.23 (16)
O1—C1—C2—C8	0.8 (2)	N1—C10—C11—C12	145.36 (13)
C6—C1—C2—C8	−179.33 (14)	C9—C10—C11—C12	−101.88 (15)
C1—C2—C3—C4	0.2 (2)	N1—C10—C11—C16	−38.83 (17)
C8—C2—C3—C4	178.85 (14)	C9—C10—C11—C16	73.92 (16)
C2—C3—C4—C5	0.6 (2)	C16—C11—C12—C13	−0.2 (2)
C3—C4—C5—C6	−0.8 (3)	C10—C11—C12—C13	175.58 (13)
C4—C5—C6—C1	0.3 (3)	C11—C12—C13—O2	−179.32 (13)
O1—C1—C6—C5	−179.62 (15)	C11—C12—C13—C14	0.0 (2)
C2—C1—C6—C5	0.5 (2)	O2—C13—C14—C15	179.53 (14)
C6—C1—O1—C7	6.1 (2)	C12—C13—C14—C15	0.2 (2)
C2—C1—O1—C7	−174.01 (16)	C13—C14—C15—O3	179.30 (13)
C3—C2—C8—N2	4.0 (2)	C13—C14—C15—C16	−0.3 (2)
C1—C2—C8—N2	−177.35 (14)	O3—C15—C16—C11	−179.44 (13)
C3—C2—C8—C9	−176.46 (14)	C14—C15—C16—C11	0.1 (2)
C1—C2—C8—C9	2.2 (2)	C12—C11—C16—C15	0.1 (2)
N2—C8—C9—C10	−7.73 (16)	C10—C11—C16—C15	−175.64 (13)
C2—C8—C9—C10	172.73 (13)	C14—C13—O2—C17	−2.7 (3)
C8—C9—C10—N1	10.06 (13)	C12—C13—O2—C17	176.6 (2)
C8—C9—C10—C11	−109.04 (12)	C16—C15—O3—C18	0.5 (2)
C11—C10—N1—C19	−67.02 (17)	C14—C15—O3—C18	−179.07 (14)
C9—C10—N1—C19	173.65 (13)	N2—N1—C19—O4	−179.14 (14)
C11—C10—N1—N2	108.85 (13)	C10—N1—C19—O4	−3.7 (2)
C9—C10—N1—N2	−10.49 (15)	N2—N1—C19—C20	0.9 (2)
C2—C8—N2—N1	−179.04 (12)	C10—N1—C19—C20	176.37 (15)
C9—C8—N2—N1	1.37 (16)		

### Hydrogen-bond geometry (Å, º)

**Table:**

*D*—H···*A*	*D*—H	H···*A*	*D*···*A*	*D*—H···*A*
C10—H10···O4^i^	0.99	2.46	3.4340 (18)	167
C5—H5···O4^ii^	0.94	2.59	3.309 (2)	134

Symmetry codes: (i) −*x*+1, −*y*+1, −*z*; (ii) *x*, *y*, *z*+1.
